# Extraction Yields of Psilocybin and Psilocin: A Short Review of Current Methods and Their Implications

**DOI:** 10.3390/ph18030380

**Published:** 2025-03-07

**Authors:** Taynah P. Galdino, Lucas C. Oliveira, Mateus A. Luz, Raquel A. Jesus, Eunice P. N. Lima, Maria C. M. Torres, Katia Sivieri, Victor I. Afonso, João M. P. Q. Delgado, Antonio G. B. Lima, Suédina M. L. Silva, Marcus V. L. Fook

**Affiliations:** 1 Northeast Biomaterials Evaluation and Development Laboratory, CERTBIO, Academic Unit of Materials Engineering, Federal University of Campina Grande, Campina Grande 58429-900, Brazil; taynah.galdino@certbio.ufcg.edu.br (T.P.G.); lucas.cordeiro@certbio.ufcg.edu.br (L.C.O.); mateus.araujo@certbio.ufcg.edu.br (M.A.L.); raquel.albino@estudante.ufcg.edu.br (R.A.J.); eunice.lima@certbio.ufcg.edu.br (E.P.N.L.); suedina.maria@professor.ufcg.edu.br (S.M.L.S.); 2 Department of Chemistry, State University of Paraíba, Campina Grande 58429-500, Brazil; mariatorres@servidor.uepb.edu.br; 3 Departament of Food and Nutrition, Faculty of Pharmaceutical Sciences, Sao Paulo State University, Araraquara 14800-903, Brazil; katia.sivieri@unesp.br; 4 Academic Unit of Physics, Federal University of Campina Grande, Campina Grande 58429-900, Brazil; viafonso@df.ufcg.edu.br; 5 CONSTRUCT-GFC, Department of Civil Engineering, Faculty of Engineering, University of Porto, 4200-465 Porto, Portugal; 6 Mechanical Engineering Department, Federal University of Campina Grande, Campina Grande 58429-900, Brazil; antonio.gilson@ufcg.edu.br

**Keywords:** extraction methods, yield optimization, psychoactive compounds

## Abstract

The growing body of evidence supporting the therapeutic efficacy of psychoactive substances, like psilocybin, has driven significant interest in recent decades due to their low toxicity and potential applications in treating various mental health disorders. However, producing pharmaceutical-grade psilocybin remains challenging, with three primary approaches: chemical synthesis, biosynthesis, and extraction from *Psilocybe* mushroom fruiting bodies. This systematic review evaluates the extraction and quantification methods for psilocybin and psilocin, aiming to contribute to the development of standardized protocols that ensure compound quality and purity. A total of 25 relevant studies were selected from an initial pool of 9152 publications indexed in platforms such as Scopus, ScienceDirect, Web of Science, and PubMed. The findings indicate that both the extraction method and the choice of mushroom species significantly influence compound yields. Ultrasonic bath extraction was identified as the most efficient technique, particularly for species including *Psilocybe cyanescens* and *Psilocybe cubensis*. High-performance liquid chromatography (HPLC) was the most-used method for identifying and quantifying these compounds. Furthermore, polar solvents were critical for effective solubilization, with parameters such as temperature, solvent-to-material ratio, and extraction time playing key roles in optimizing yields. This review serves as a key scientific reference for advancing research, enhancing analytical precision, and ensuring reproducibility through the standardization of extraction and quantification protocols.

## 1. Introduction

The use of plants and mushrooms capable of inducing altered states of consciousness in ritual contexts has been documented across numerous cultures and societies throughout millennia of human history. Psilocybin, a psychoactive indole alkaloid (tryptamine), is found in over 100 species of fungi within the *Psilocybe* genus, commonly referred to as “magic mushrooms”. Psilocybin acts as a prodrug of psilocin, which, upon ingestion, induces alterations in subjective perception, thought processes, emotions, and cognition. These mental effects may include euphoria, heightened feelings of happiness and peace, physical and mental relaxation, enhanced mental clarity, altered perceptions of time and space, disconnection from the environment, distortions in the perception of size, and experiences described as unreal or otherworldly [1,2].

In the mid-20th century, following Albert Hofmann’s synthesis of psilocybin in 1958, it and other classical psychedelics (for example LSD, DMT, and mescaline), which act as agonists of the 5-hydroxytryptamine2A (5-HT2A) GPCR neuroreceptor, became central to psychiatric research as experimental therapies. However, the widespread use of these substances outside controlled medical contexts eventually led to their prohibition, resulting in a near-complete cessation of scientific research at the time. Specifically, both psilocybin and psilocin were classified as Schedule I substances under the 1971 United Nations Vienna Convention on Psychotropic Substances. Consequently, research involving these compounds remains highly regulated according to national standards. In Brazil, for example, studies involving the manipulation of these substances require special authorization from the National Health Surveillance Agency (ANVISA, Brasília, Brazil), the counterpart to the US Food and Drug Administration (FDA, Silver Spring, MD, USA).

Despite strict legal restrictions, recent decades have seen numerous studies reaffirm earlier findings while uncovering new evidence highlighting the therapeutic potential of psilocybin and psilocin in addressing various mental health conditions. These include alcohol and tobacco addiction [3], major depressive disorder (MDD) and treatment-resistant depression (TRD) [4,5,6], end-of-life depression and anxiety [7,8], post-traumatic stress disorder (PTSD) [9], obsessive–compulsive disorder (OCD) [10,11], eating disorders like anorexia nervosa [12], and other conditions currently under investigation. Amid a global mental health crisis, the renewed interest in psychedelics as therapeutic agents has fueled both social and scientific enthusiasm, leading to a notable surge in related scientific publications [13].

Furthermore, for clarity, mental health conditions encompass mental disorders, psychosocial disabilities, and other mental states that are associated with significant distress, functional impairment, or a risk of self-harm. In 2019, prior to the COVID-19 pandemic, an estimated 970 million people worldwide were living with a mental disorder, with anxiety and depression being the most prevalent [14]. Specifically, while the precise mechanism of action is not yet fully understood, psilocin has been suggested as a potential modulator of functional connectivity across different brain regions [15]. This implies a promising therapeutic potential for a range of neuropsychiatric disorders.

Within the human body, psilocybin undergoes metabolic transformation, beginning with dephosphorylation in the intestinal mucosa, a process catalyzed by alkaline phosphatases. This conversion transforms psilocybin into its psychoactive form, psilocin. Psilocin is subsequently absorbed into tissues, where it exerts its pharmacological effects [16].

Structurally similar to serotonin, psilocin has been shown to activate several receptors within the central nervous system, including serotonergic 5-HT receptors (and the serotonin transporter, SERT), dopaminergic (D) receptors, imidazole (I) receptors, and α-adrenergic receptors [17]. The ability of these substances to modulate serotonergic and glutamatergic activity has demonstrated potential therapeutic effects [18], for example, antidepressant and anxiolytic actions. Additionally, some studies [19,20,21] have suggested that psilocybin may influence brain function by promoting neuroplasticity, enhancing neural connectivity, stimulating neurogenesis, reducing inflammation, and improving cognition. These mechanisms may offer potential benefits for addressing mental health conditions linked to disrupted neural pathways and other underlying issues [22,23,24].

Mood disorders, including depression, post-traumatic stress disorder (PTSD), and anxiety, are associated with low-grade chronic neuroinflammation, detectable through peripheral inflammatory markers such as TNF-α. Psilocybin exhibits specific anti-inflammatory effects by downregulating pro-inflammatory mediators like TNF-α and IL-1β, thereby mitigating low-grade neuroinflammation, restoring altered neural connectivity in depression, facilitating the reprocessing of traumatic memories, reducing amygdala hyperactivity, and enhancing emotional resilience in patients with anxiety and PTSD. Additionally, psilocybin contributes to addiction treatment by disrupting maladaptive behavioral patterns, often linked to profound insight experiences and enhanced mental clarity [25,26,27].

The growing body of evidence supporting the potential efficacy of these psychedelic substances, coupled with their exceptionally safe profile for human use in medical contexts, well-documented pharmacological effects, and very low potential for abuse, provides a strong incentive for further research into these novel therapeutic agents. These effects are enhanced when the substance is administered in a controlled therapeutic setting with appropriate psychological support, highlighting its potential as a transformative intervention for complex mental health conditions. In this regard, the FDA’s recognition of psilocybin’s therapeutic potential in 2018, demonstrated by the granting of the “Breakthrough Therapy Designation” (BTD) for its use in treating major depressive disorder (MDD) [28], marked a significant milestone. In 2024, the BTD was also granted to a patented deuterated psilocybin analog (Cybin’s CYB003) for the adjunctive treatment of MDD, further reinforcing the confidence and optimism within the scientific community.

On the other hand, a series of legal reforms in the United States and Canada, including the decriminalization of psychedelic mushrooms [29] and psilocybin [30], as well as the legalization of therapeutic psilocybin use in certain US states/cities [31] (with others considering similar measures), have paved the way for legal security. Additionally, Australia recently regulated the prescription of psilocybin by licensed psychiatrists for the treatment of post-traumatic stress disorder (PTSD) [32]. These changes have naturally spurred commercial interest and significant investment, with projections suggesting that the psychedelic substances market could reach USD 10 billion by 2027 [33]. Accordingly, an increasing number of proprietary techniques for producing pharmaceutical-grade psilocybin have been patented in recent years [34].

The advancement of psilocybin-related clinical trials has led to a growing demand for reliable, pharmaceutical-grade psilocybin. It is essential for physicians to have confidence in the product’s consistency, stability, and quality to ensure that patients receive safe, consistent, and effective doses. As such, any method employed to obtain psilocybin must be scalable and capable of producing a consistent, pure, and stable product [35].

There are three primary methods for obtaining psilocybin: traditional chemical synthesis [35,36,37,38], the more recent biosynthetic pathway [39,40], and cultivation with natural extraction from fungal fruiting bodies. Regarding the latter, to the best of the authors’ knowledge, no recent publications provide a comprehensive overview of the various extraction and identification methods for the fungi of the *Psilocybe* genus. Therefore, the aim of this article is to offer a review that enhances the understanding of the existing methods of extraction, identification, and quantification of *Psilocybe* hallucinogenic fungi, with the hope it may serve as an important initial step toward developing standardized protocols for the extraction, analysis, and quality control of these promising substances for human health.

## 2. Methodology

### 2.1. Elegibility Criteria

#### 2.1.1. Inclusion Criteria

This systematic review utilized the PICO framework (Population, Intervention, Comparators, and Outcome) to establish the inclusion criteria, following the methodology proposed by Methley, Campbell [41]. Eligible studies were those that quantified and reported the highest yields of psilocybin and psilocin extraction (outcome) using various extraction techniques (intervention). Additionally, the studies compared different methodologies and operational parameters (comparators) across a range of psychedelic mushroom species (population).

#### 2.1.2. Exclusion Criteria

The exclusion criteria applied to this systematic review were as follows: (i) studies reporting psilocybin and/or psilocin yields from synthetic sources; (ii) investigations involving fractionated extracts or isolated psilocybin compounds; (iii) studies that did not quantify the substances; (iv) research quantifying these substances in biological tissues or fluids; and (v) review articles, book chapters, theses, letters, personal opinions, conference abstracts, and patents.

### 2.2. Information Sources and Search Strategy

A literature review was conducted by creating specific search strings tailored for each bibliographic database consulted, including PubMed, Engineering Village, Sage Journals, ScienceDirect, Scopus, and Web of Science (see Table A1). The search was carried out in 2024, with no restrictions on language or publication dates. Duplicate references were removed by using the reference management software StArt (version 3.4 BETA) (LaPES, São Carlos, SP, Brazil).

### 2.3. Study Selection

The article selection process was carried out in two distinct phases to ensure that each study met the predefined inclusion and exclusion criteria. In the first phase, two authors (T.P.G. and E.P.N.L.) evaluated the titles and abstracts of all studies retrieved from the search. In the second phase, three independent pairs of authors (T.P.G. and E.P.N.L.; L.C.O. and M.A.L.; and M.C.M.T. and R.A.J.) reviewed the full texts of the studies that passed the initial screening, excluding those that did not meet the inclusion criteria (see Table A2). After the selection process was completed, relevant data from the included studies were extracted independently by each pair. Any discrepancies between the pairs were resolved through discussions with a third author.

### 2.4. Quality Analysis in Individual Studies

The quality assessment of the studies included in this review was conducted using the Joanna Briggs Institute (JBI) Critical Appraisal Tool [42], which was modified to suit methodological studies performed in laboratory settings. This tool was chosen for its comprehensive approach to evaluating the methodological rigor and reproducibility of experimental studies. Each question was independently addressed by two reviewers, with any discrepancies resolved through consultation with a third reviewer. Responses were categorized as high for studies that answered the questions, low for those that did not answer the question, unclear when assessment was not possible, no information if relevant data were missing, and not applicable if the question was not relevant to the type of study under review.

## 3. Results and Discussion

### 3.1. Study Selection

The selection of studies was based on the construction of a search string designed to retrieve articles relevant to the extraction and quantification of psilocybin and psilocin. The general search string, covering the English, Portuguese, and Spanish languages, was as follows: “(Psilocybin OR Psilocibina OR Psilocybina OR Psilocin OR Psilocina) AND (Extraction OR Extração OR Extracción OR Identification OR Identificação OR Identificación OR Characterization OR Caracterização OR Descripción OR Quantification OR Quantificação OR Cuantificación)”. This search resulted in a significant number of articles identified across several databases. On 11 September 2024, the application of this string on the Scopus platform, with adaptations for each database (see Table A1), yielded a total of 3843 publications. A previous search in January 2022 using the same string on the same database returned 2390 studies, highlighting a significant increase in the number of publications and reflecting growing academic and industry interest in psilocybin and psilocin.

**Figure 1:**
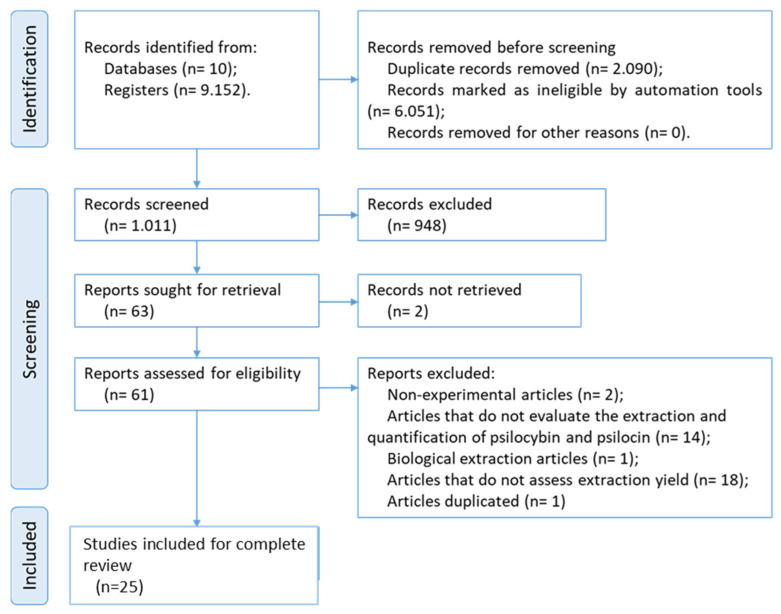
Flow diagram of the literature search and selection criteria from PRISMA [43].

By expanding and refining the search for new articles related to the topic under study across multiple platforms and using similar terms, additional results were obtained (see Table A1). In this systematic review, as shown in Figure 1, a total of 9152 studies were identified across several databases, including 3882 from Scopus, 3065 from ScienceDirect, 818 from Sage Journals, 195 from Web of Science, 253 from BVS, 253 from LILACS, 332 from PubMed, 293 from Embase, 39 from Cochrane, and 22 from Engineering Village. After using the StArt tool (version 3.4 Beta) (LaPES, São Carlos, SP, Brazil) to remove duplicates (2090 articles) and applying the minimum score involving the identification of the main terms in the title, abstract, and keywords (6051 studies), 1011 studies remained. A subsequent reading and evaluation of the “title and abstract” led to the exclusion of 948 articles. Among the 63 selected for full reading based on predefined criteria (see Table A2), 25 studies were chosen for systematic evaluation.

### 3.2. Characteristics of the Included Studies

**Figure 2:**
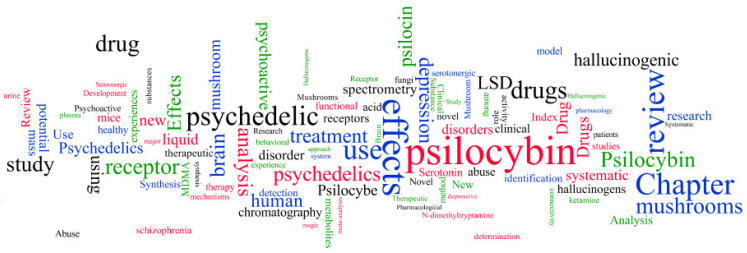
Co-occurrence of terms found in “Titles, Keywords and Abstracts” of the 25 publications included in the systematic review. Figure generated through the occurrence of the most used terms identified by the StArt software (version 3.4 BETA).

All of the studies included in this review were articles that evaluated an extraction process for psilocybin and psilocin, quantified these compounds, and enabled the calculation of extraction yields. Regarding the references cited in these documents, Figure 2 presents a word cloud diagram in which various terms appear in different font sizes, representing their frequency of occurrence across the 25 academic articles analyzed. Larger font sizes indicate the most frequently encountered terms, highlighting key topics such as psilocybin, which is more commonly analyzed than psilocin, along with its characteristics and effects. Conversely, smaller words represent less-frequent terms, arranged organically without strict alignment, creating a dynamic visual effect. The figure also includes references to extraction methods and chromatographic analysis for the quantification of these compounds, though to a more limited extent.

The studies analyzed span a long period, from the late 1970s to the current year of the search, with interest being rekindled in the last two decades. Recent peaks in publications suggest an increased interest in the topic, likely driven by the growing body of research on the potential therapeutic effects of psilocybin.

The studies cover various extraction methods, ranging from ultrasonic bath (*n* = 9), agitation methodologies (*n* = 6), maceration (*n* = 3), shaker (*n* = 2), combined methods (*n* = 4), and others not displayed (*n* = 1). In 15 of the reviewed studies, the focus was on the quantification and characterization of psilocybin and psilocin, primarily using chromatography and mass spectrometry. Techniques such as gas chromatography (GC) and high-performance liquid chromatography (HPLC) are the most common, offering greater precision in quantifying these compounds. Additionally, recent studies have incorporated DNA identification methods, which improve the accuracy of taxonomic identification of mushroom species.

Six studies specifically focus on forensic and toxicological applications, aiming to identify and quantify hallucinogenic compounds and assess cases of intoxication. This line of research is crucial for providing regulators and healthcare professionals with data on the risks of uncontrolled consumption of psychoactive mushrooms, highlighting the importance of the topic in legal and forensic contexts. Accurate identification of psychoactive compounds helps monitor and control mushroom use.

Although extraction and analysis techniques have evolved, some studies still present methodological limitations, for instance, the difficulty in standardizing compound extraction due to variability between mushroom species. This underscores the need for an analysis of each parameter employed, with the aim of identifying consistencies between the implemented methodologies and seeking to standardize extraction methods that yield the highest amounts of psilocybin and psilocin.

### 3.3. Quality of Individual Studies

**Figure 3:**
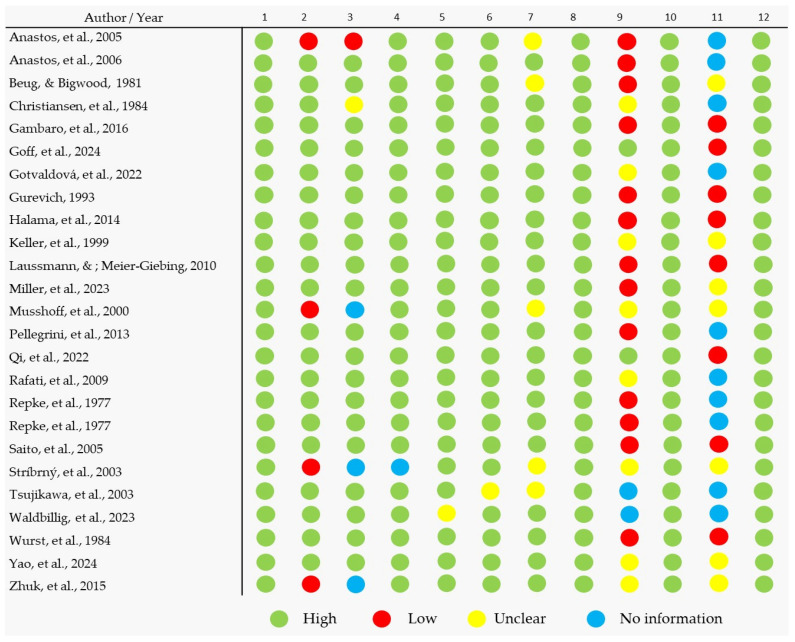
Overall quality of the selected studies [35,40,44,45,46,47,48,49,50,51,52,53,54,55,56,57,58,59,60,61,62,63,64,65,66]. A detailed description of the evaluated parameters can be found in Table A3.

The quality assessment was conducted based on guidelines adapted from the Joanna Briggs Institute (JBI) Critical Appraisal Tool, addressing the questions modified for the critical analysis of experimental articles for all included studies (Figure 3) with predefined criteria (Table A3).

The analysis of the methodological quality of the 25 evaluated articles, based on 12 specific criteria (Figure 3 and Table A3), reveals important considerations regarding the scientific rigor in the field of psilocybin protection and quantification. From the obtained data, it is observed that, in general, the studies have clear objectives aligned with the central theme, with the majority being classified as high quality (represented by the green color) in these evaluations. This characteristic highlights the maturity of many studies in establishing scientific relevance and practical applicability in the field of psilocybin. However, a detailed analysis exposes the methodological gaps that compromise the replicability and reliability of some of these studies, pointing to areas that require greater technical rigor.

A widely noted positive aspect was the detailed description of extraction methodologies, with most articles outlining variables like solvents used, removal times, and temperature conditions. These details are crucial, as they ensure not only reproducibility but also allow for direct comparisons between studies. However, in the few cases where methodologies were assessed as insufficiently described (marked in red), this flaw undermines the ability to validate the results presented, highlighting the need for greater standardization in reporting experiments. The sample preparation process, including steps like drying, grinding, and storage, was also satisfactorily reported in most articles. However, the absence of clear information in some studies highlights an area for improvement, as these steps significantly influence the results obtained in the processes.

The criteria related to the justification of solvents and reagents used, as well as the efficiency of the extraction process (yield of psilocybin and psilocin), showed greater variability in quality. Studies that justified their choices aligned more strongly with the objectives of the process, allowing for a more reliable assessment of the results obtained. On the other hand, the lack of clear justifications or the absence of data related to extraction yields in some articles (marked as unclear or no information) limits the practical utility of the methodologies presented. These issues highlight a significant gap in the field: the absence of standardized benchmarks for evaluating efficiency, which complicates the comparison between different protocols.

An analysis of the analytical methods for quantifying psilocybin, including HPLC, TLC, and GC-MS, revealed significant discrepancies in the validation and suitability of these techniques. While many studies robustly validated their methods, a specific subset lacked sufficient information on the reliability of the analytical methods employed. The absence of rigorous validation undermines the accuracy of results, which is a critical aspect considering the impact of psilocybin in clinical and pharmaceutical studies. Furthermore, the reproducibility of experiments, a fundamental principle in experimental science, was frequently observed as one of the criteria with the highest number of LOW and UNCLEAR evaluations, highlighting the methodological weaknesses. This undermines not only the reliability of individual results but also the collective advancement of the field.

The presentation of the data and the results was generally clear and organized, with statistical analyses assessed in high-quality studies. However, discussions about methodological limitations and challenges, as well as potential sources of bias, were often understated. Many studies did not critically address these issues, reflecting a possible lack of reflection on the robustness and applicability of the results obtained. This undermines scientific transparency and reduces confidence in the generalizability of the findings.

In summary, the results reveal a promising scenario but still present significant challenges. While many studies demonstrate high quality in essential criteria, such as, for example, in defining objectives and methodological procedures, gaps in reproducibility, analytical validation, and critical discussion highlight the need for greater scientific rigor, the adoption of standardized methodological guidelines, the analysis of parameters, and the consideration of variable influences, as well as the promotion of open science practices, could significantly contribute to strengthening the field of psilocybin extraction and quantification, fostering more reliable advancements.

We recommend the use of ultrasonic-assisted extraction (UAE) as the most effective technique, given its ability to maximize yield through cavitation effects that promote efficient cell wall disruption. The optimal solvent for psilocybin extraction is methanol acidified with acetic acid or hydrochloric acid, which has been shown to enhance compound solubility while preventing psilocin degradation. The recommended solvent-to-material ratio is at least 1:100 (*m*/*v*), as higher ratios improve compound recovery.

To minimize variability, temperature should be maintained between 20–25 °C, preventing thermal degradation of psilocin while ensuring adequate diffusion. Extraction time should be at least 30 min, with the possibility of extending up to 3 h, depending on the mushroom species and solvent system used. For enhanced efficiency, we also recommend multiple consecutive extractions, as this approach increases compound recovery while maintaining sample integrity.

Furthermore, for analytical quantification, high-performance liquid chromatography (HPLC) coupled with UV or mass spectrometry detection should be the standard technique, ensuring precise identification and quantification of psilocybin and psilocin. Additionally, incorporating chemometric approaches and multivariate analysis can help optimize extraction parameters and identify the most influential variables affecting yield and reproducibility.

### 3.4. Extraction Techniques of Psilocybin and Psilocin in Hallucinogenic Mushrooms

The extraction process is critical for obtaining psilocybin and psilocin alkaloids from mushrooms, as it directly influences the yield and purity of these psychoactive compounds [67,68]. The selection of an appropriate extraction method is based on the properties of the metabolites and the solvent’s ability to recover the maximum amount and concentration of the target compounds, without altering their chemical structure [69]. Historically, since the 1950s, polar solvents like methanol, ethanol, and hydroalcoholic solutions have been used to extract psilocybin, due to its high polarity, which is attributed to the presence of the phosphate group, while psilocin, being less polar, is slightly more soluble in nonpolar solvents. Various factors impact the efficiency of the extraction, including the species of mushroom, the ratio of fungal mass to solvent, and the extraction technique employed. Common extraction methods include maceration, Soxhlet extraction, reflux extraction, magnetic agitation, and ultrasound-assisted extraction [70,71,72,73]. Each technique offers distinct advantages, including but not limited to higher yields or better control over temperature and extraction time, which are crucial for ensuring the recovery of the target compounds in their natural, unmodified form.

Another important factor to consider when analyzing the content of psilocybin and psilocin in mushrooms is the genus and species, as different species naturally produce varying percentages of these compounds. The *Psilocybe* genus is particularly prominent in this regard due to its wide distribution across multiple continents, the variety of species it encompasses, and its extensive history of research. This makes *Psilocybe* species ideal candidates for further studies on psilocybin and psilocin, with a well-established body of knowledge to support future investigations [61]. When extracting these metabolites, different parts of the mushroom can be utilized, either individually or by combining various portions, to determine the most effective method for obtaining the highest yields. Table 1 as referenced summarizes the species of mushrooms from various fungal genera that have been used to extract psilocybin (PSCB) and psilocin (PSC), along with the specific mushroom parts employed and the extraction methods utilized, and the corresponding yields obtained. This information serves as a valuable reference for understanding the diversity of methods and their respective yields across different mushroom species.

Following the presentation of these findings, a detailed discussion on the types of mushrooms used, the extraction methods employed, and the yields obtained is essential to assess the effectiveness and optimization of these techniques.

(a) Regarding the mushroom’s parts, among the papers reviewed, one focused on the extraction of psilocybin and psilocin from the mushroom cap, one study investigated the sclerotia, four employed both the cap and stem, none exclusively utilized the stem, eleven studies extracted from the entire mushroom, and finally, two studies assessed the cap, stem, and whole mushroom for extraction.

Regarding the various methodologies and species of mushrooms used for the extraction of psilocybin and psilocin, it is observed that the whole mushroom is predominantly used, although the cap appears to yield higher results when compared to the stem and the whole mushroom, as shown in the study considering *Psilocybe cubensis* [55]. According to that reference, the yield values for the cap ranged from 0.44–1.35% and 0.17–0.78%, while for the stem, these values were slightly lower, ranging from 0.05–1.27% and 0.09–0.9%, respectively. In the species *Psilocybe ovoideocistidiata* [57] and *Psilocybe pelliculosa* [58], the authors obtained psilocybin and psilocin yields ranging from 1.02 to 1.79% and 0.14 to 0.46% for the cap, and from 0.17 to 0.19% and approximately 0.04% for the stem, respectively, indicating that both parts have bioactive potential, but with varying concentrations, confirming that the cap may contain a higher concentration of the target compounds for the same methodology studied.

In fact, the cap tends to have higher levels of psilocybin and other metabolites than the stem due to biological and structural factors. Theories aim to justify these factors, citing localized biosynthesis, the defensive function of alkaloids, and different cellular structures. The caps of the mushrooms have a cellular organization that facilitates the production and storage of psychotropic compounds such as psilocybin, along with other metabolites that play a role in protecting the mushroom, being responsible for the production and dispersion of spores. In comparison, the stem has a higher proportion of structural tissues, including fibers and chitin, with less space for the storage of secondary metabolites [62,74,75,76].

By evaluating the yields reported in the analyzed articles, it is observed that psilocybin is present in greater quantities than psilocin, with yields ranging from 0–4.13% and 0–1.77%, respectively. This difference may be attributed to the greater chemical stability of psilocybin compared to psilocin, which is more prone to degradation, particularly under less controlled environmental conditions involving oxygen, heat, and light [49,77].

Several factors can influence the percentages of psilocybin and psilocin present in mushrooms, ranging from environmental factors to variations in the extraction process. Among the environmental factors, the cultivation substrate, mushroom maturity, environmental conditions (temperature, relative humidity, and lighting), species genetics, and intraspecific variability can be highlighted. The cultivation substrate and pH significantly affect yields, directly influencing alkaloid synthesis. Mushroom maturity and environmental conditions are also crucial, as mushrooms harvested at full maturity tend to exhibit higher concentrations of psilocybin. In addition, temperature and humidity control optimize production, while light is essential to induce fruiting [69,78,79].

The species that showed yields above 1.5% psilocybin include *Panaeolus subbalteatus* [50] (4.13% extracted from the cap, 1.90% from the stem), followed by *Psilocybe cyanescens* [53] (3.42%), [61] (1.57%), and [49] (1.38%); *Psilocybe ovoideocistidiata* [57]; *Psilocybe pelliculosa* [58] (1.79%); *Psilocybe semilanceata* [66] (1.58%); and *Psilocybe serbica* var. *bohemica* [49] (1.55%), with both obtained from the extraction of the whole mushroom. For psilocin, percentages above 1% were only identified in *Psilocybe cyanescens* [53] (1.19% extracted from the whole mushroom).

(b) Regarding the mushrooms’ genus/species, among the 25 articles analyzed in this review, 62 mushroom species were identified across the various genera used for compound extraction, demonstrating significant variations in extraction yields. The most commonly utilized genus for extraction was *Psilocybe* (24 species), with *Psilocybe cubensis* being the most frequently used species (approximately 30% of the articles), which may be related to its traditional use, ease of cultivation, and abundance of psychoactive and therapeutic compounds, namely psilocybin and psilocin. The second most commonly used species was *Psilocybe semilanceata* (approximately 25%), followed by the genus *Panaeolus* (11 variations), which presented distinct extraction yields depending on the methodologies and parameters employed.

Species of the *Psilocybe* genus are widely known for their high psilocybin content, with concentrations varying significantly among different species and extraction methodologies. Mushrooms of the species *Psilocybe cubensis* and *Psilocybe cyanescens* tend to grow in distinct habitats, while *P. cubensis* is often found in tropical and subtropical regions. *P. cyanescens* is common in temperate and humid regions. Comparatively, the genus *Panaeolus*, specifically *Panaeolus subbalteatus*, is found in a wide range of habitats, including pastures and manure-rich locations, which may influence the production of secondary compounds like psilocybin, similar to what is observed in *P. cubensis* [80]. In contrast, other genera, such as Inocybe and Conocybe, produce significantly lower levels of these compounds. This may be related to the chemical and ecological evolution of these species, which might not use the same biosynthetic mechanisms or may lack selective pressures to develop high concentrations of psychoactive compounds. These ecological factors can influence the biosynthesis of the compounds, as alkaloid production may be linked to defense against herbivores or pathogens in specific environments [77,78]. Furthermore, the evaluation of the extraction methodologies and parameters provides a convincing basis for the higher extraction yield of these compounds, since, for the same mushroom species, the authors obtained yields with variability above 30%.

(c) Regarding the extraction method, several extraction methods have been attempted for secondary metabolites from mushrooms. These methods include maceration, Soxhlet extraction, mechanical/magnetic stirring, ultrasound, supercritical fluid extraction (SFE), and solid-phase microextraction (SPME), with considerations regarding the efficiency, purity, time, cost, and safety of each methodology. Supercritical fluid extraction and solid-phase extraction techniques are not mentioned in the articles analyzed, probably because, despite their high efficiency and purity, these methods require very specific equipment or produce limited quantities of material, and their yields are not calculated, making them unsuitable for use in reasonably general conditions.

The most frequently applied methodologies for the extraction of psilocybin (PSCB) and psilocin (PSC) include ultrasonic bath (approximately 35% of the studies), agitation (with an emphasis on vortex use), and maceration. The ultrasonic bath presents higher yields for both substances in species like *Psilocybe cyanescens*, with psilocybin concentrations ranging from 1.61% to 3.42% and psilocin from 0.62% to 1.77% [53]; *Panaeolus cyanescens*, where values between 0.02% and 1.15% for PSCB and between 0.14% and 0.9% for PSC were obtained [55]; and the species *Pholiotina cyanopus*, yielding PSCB values from 0.82% to 0.98% and PSC from 0.16% to 0.18% [51]. The ultrasonic bath method utilizes the cavitation process to efficiently break down cell walls, releasing bioactive compounds. Cavitation promotes the formation and implosion of microbubbles that increase the exposure of the compounds to the solvent, and high-pressure waves provide rapid release of the compounds, resulting in high yields, particularly in species that are rich in indolic alkaloids, especially psilocybin.

Vortex agitation is another widely used method, especially in species such as *Psilocybe cyanescens*, yielding PSCB between 0.23% and 1.38% and PSC between 0.04% and 1.00%, and *Psilocybe serbica*, with PSCB ranging from 0.15% to 1.55% and PSC from 0.002% to 0.038% [49]. Although the yield is lower than that obtained with an ultrasonic bath, vortex agitation still allows for effective extraction in species with less resistant cell walls.

In the extraction using maceration, the results generally provide lower yields, likely due to the characteristics of the method being less aggressive and slower. However, it can be optimized with a prolonged use of solvents and appropriate temperatures. This method has proven efficient in species including *Panaeolus subbalteatus*, with PSCB yields ranging from 1.10% to 1.30% and no detectable PSC [50], and *Psilocybe subaeruginosa* [44], suggesting that prolonged solvent diffusion can extract significant amounts of compounds in more permeable species.

Corroborating the data identified by the yield analysis of only the mushroom parts, regardless of the extraction technique implemented, the cap tends to exhibit slightly higher concentrations, highlighting the influence of the concentration of enzymes and metabolites involved in the biosynthesis of psychoactive alkaloids. Thus, it is evident that the extraction method directly influences the efficiency of obtaining psilocybin and psilocin due to physicochemical factors that affect the release of the compounds from the mushroom cells. Moreover, among the methods evaluated, the ultrasonic bath proved to be the most effective for extracting these substances, achieving higher yields in several species, especially *Psilocybe cyanescens*, *Psilocybe cubensis*, and *Panaeolus cyanescens*. The cavitation generated by ultrasound proved efficient for mushrooms with dense cellular structures, while vortex agitation was effective in species with less rigid cell walls, yielding amounts that can exceed 1.5% for psilocybin and 1.0% for psilocin. Maceration, although less aggressive, produced good results in some species with more malleable cellular structures, reinforcing the importance of choosing the appropriate extraction method and considering the characteristics of the species and the mushroom part to maximize the yields of psilocybin and psilocin.

After analyzing the extraction methodologies, the next step involved a detailed evaluation of the specific parameters for each technique, as shown in Table 2. This step became crucial for understanding how the variables of extraction time, temperature, ultrasound frequency, and agitation speed directly influence the yield and efficiency of psilocybin and psilocin release. Understanding these parameters not only allows for the optimization of each methodology but also helps identify the ideal conditions for each species and part of the mushroom, providing a more efficient and economically viable extraction.

Considering only the extraction methodologies and parameters, the whole mushroom was used to evaluate the yield of psilocybin and psilocin in relation to different parameters such as methodology, mushroom form, pulverization technique, solvent type, proportion, time, number of extractions, and temperature. The use of powdered mushrooms is predominant in the studies, with pulverization facilitating the release of compounds during extraction. The pulverization technique, whether using a mill or mortar and pestle, seems to influence the yield less compared to other factors, although the use of a mill is common in combinations of vortex agitation and ultrasonic bath extraction methods, possibly to ensure greater sample homogeneity.

Among the methodologies described and explored, those that used the ultrasonic bath tend to generate higher yields [51,55,60,62,66]. Furthermore, combining this methodology with agitation, such as using a mixer, enhances the yield, reaching up to 3.422% for PSCB and 1.767% for PSC [53]. However, in specific cases [50], maceration with a water and ethanol solvent can generate high yield values for PSCB (1.1–1.9%), demonstrating the importance of choosing the appropriate methodology, solvent, and process variables.

(d) Regarding the extraction solvent, in extracted materials, with an emphasis on alkaloids, the choice of solvent type significantly affects the quantity and purity of the extracted compounds. Among the solvents used, methanol, methanol with additives, and, less frequently, water and ethanol were the main ones selected by the authors, exhibiting specific characteristics that favor the composition of polar and semipolar compounds, which are crucial attributes for understanding the yields obtained.

Methanol, with or without additives including acetic acid, ammonium nitrate, hydrochloric acid, and formic acid, is the most commonly used solvent (approximately 75%) in various references [40,47,53,54]. This solvent is frequently used due to its strong affinity for polar compounds, such as psilocybin, and its ability to cross plant cell walls and facilitate the release of intracellular compounds. Furthermore, it acts to protect temperature- and oxidation-sensitive compounds, like psilocin, during longer degradation processes. The addition of acids or other reagents can help modify the pH and improve the solubility of target compounds, increasing the extraction yield, as well as contributing to the stabilization of the extracted molecules, preventing oxidation and thereby enhancing the yield.

Among the articles analyzed, extractions performed exclusively with methanol showed PSCB yields ranging from 0.0% to 1.56%, and PSC yields between 0.04% and 1.58% [47,48,56,61,66], reflecting methanol’s ability to effectively interact with psilocybin, facilitating the solubilization of polar compounds. It should be noted that the highest yield of psilocin with methanol (1.58%) was obtained for a single species, extracted using the Shake method, and was not reproduced for the other species evaluated in the study, despite considerably high yields, necessitating a careful assessment of the reproducibility of this methodology. Thus, it is understood that the application of methodologies using methanol can create conditions to achieve up to 1% psilocybin extraction, except in special cases [55].

The addition of acidic solvents and salts to methanol, such as acetic acid, ammonium nitrate, or 3-indoxyl phosphate disodium, can increase the extraction efficiency of psilocybin and psilocin. The presence of acetic acid improves the stability of compounds like psilocin, which is more prone to oxidative handling, providing an acid-friendly environment and limiting the formation of undesirable by-products. For instance, in the extraction with methanol, followed by a second extraction with methanol/acetic acid and vortex agitation, the yields of PSCB ranged from 0.0002% to 1.5543%, and PSC from 0% to 1.0018%, whereas, with the use of methanol/hydrochloric acid, the yield ranged from 0.00048% to 3.422% for psilocybin and from 0.015% to 1.767% for psilocin [49].

The addition of water to solvents is an effective strategy to increase the polarity of the solvent, promoting better solubilization of highly polar compounds, while methanol or ethanol act as stabilizers to prevent compound degradation. Combinations of methanol/water [54,57,65] and water/ethanol [50] were tested, but the yields obtained were lower than those analyzed under other process conditions. Furthermore, chloroform was used in one of the extractions as an alternative solvent [35,52]. However, it was tested only in mushroom fractions, and the yields of PSCB and PSC were not significantly increased, indicating that this solvent is not suitable for the extraction of psilocybin and psilocin. The use of chloroform is concentrated on highly apolar compounds, which limit their ability to extract alkaloids such as psilocybin. This result reinforces the need for the use of polar solvents to efficiently obtain compounds.

(e) Regarding the process variables, in addition to the extraction solvents, there are other parameters that influence the yields of psilocybin and psilocin. Among the process variables, examining the influence of the material–solvent ratio, time, number of extractions, and temperature used is crucial to understanding how these factors interact to maximize the efficiency of the extraction process.

The material–solvent ratio determines the amount of solvent available to penetrate the plant material and dissolve the compounds of interest, while the contact time of the solvent influences the dissolution profile of the target substances. In the case of methanol extractions, it is observed that higher solvent ratios (1:100 and above) tend to increase the yields of PSCB compared to smaller ratios. Using a 1:50 ratio [56], the yield of psilocybin ranged from 0.10% to 0.76%, while for the 1:100 ratio [59], this parameter ranged from 0.923% to 1.379%. The higher solvent ratio, by increasing the volume of methanol available to solubilize the compounds, improves the contact between the solvent and the plant material, facilitating the protection of residual compounds that are not completely released in smaller ratios. However, excessive solvent increase does not guarantee a proportional increase in the yields due to solvent saturation. Ratios of 1:200 using vortex agitation and ultrasonic bath methods resulted in PSCB yields ranging from 0.95% to 1.03% [63].

Extraction time is a variable that, along with the methodology, determines the speed and quantity of compounds extracted. Evaluating the same methodology (ultrasonic bath and methanol) and ratio (1:100) at different times, it is observed that short times of 0.5 to 1 h resulted in moderate yields of 0.08% to 0.22% of PSCB [62], while prolonged times of 3 h resulted in yields of up to 0.98% [51], which can be justified by the increased contact time between the solvent and the material, allowing for more compounds to be distributed and extracted. Additionally, repeated extractions, with solvent replenishment, contribute to greater efficiency in the removal of psilocybin and psilocin, as each new extraction allows the recovery of residual compounds.

Temperature is a key parameter in the yields of compounds like psilocybin (PSCB) and psilocin (PSC), as it directly influences the solvent diffusion rate, compound solubility, and the stability of the extracted molecules. For most of the studies conducted, the temperature was not specified, leading to the assumption that it was ambient (20–25 °C). This choice is suitable for thermosensitive compounds like psilocin, which can degrade at higher temperatures. Extractions performed at ambient temperature include vortex renewal, renewal, and maceration methods, and show varied yields, with variations in the solvent–material ratio and time.

It is observed that the use of methanol, combined with an ultrasonic bath and elevated temperatures (around 50 °C), optimizes the yields. In the study that used an ultrasonic bath at 50 °C with methanol and a 1:90 ratio, the authors found that the temperature and two consecutive extractions provided an ideal environment for cell rupture and compound solubilization, resulting in an increased recovery of PSCB and PSC. In contrast, the study that used an ultrasonic bath at an ambient temperature with methanol and a 1:100 ratio achieved similar yields with three consecutive extractions, demonstrating that, in physical conditions where the temperature is not controlled, increasing the number of extractions proved effective in compensating for the limitations of cavitation, reduced solubility, and minimizing thermal degradation effects, although the yields remained slightly lower than those obtained under controlled temperature conditions.

Based on the analysis of the various extraction conditions, it can be stated that the yield of PSCB and PSC depends on the optimized combination of methodology, solvent–material ratio, temperature, and number of extractions. Active methodologies (e.g., ultrasonic bath) combined with high temperatures and elevated solvent ratios, along with multiple extractions, formed an efficient combination and provided the highest and fastest yields for both substances. On the other hand, passive methodologies, like maceration, require prolonged times and ambient temperature, being particularly effective for extracting psilocybin with lower degradation rates. These observations indicate that the choice of methodology and the optimization of all parameters are crucial for maximizing yields effectively and tailored to the target compound, with the need to adjust operational conditions based on the physicochemical characteristics of the extracted substances and the solubility profile of the solvents used.

The extraction of psilocybin and psilocin from fungal sources presents significant challenges, particularly concerning reproducibility and standardization. Several factors contribute to inconsistencies in reported yields across different studies, including variations in mushroom species, extraction methodologies, solvent selection, and operational parameters. Understanding these limitations is crucial for developing more reliable protocols that ensure a consistent recovery of these psychoactive compounds.

One of the major challenges in psilocybin extraction is the intrinsic variability in the chemical composition of Psilocybe mushrooms. Different species and even individual specimens within the same species can exhibit significant fluctuations in alkaloid content due to genetic diversity, environmental conditions, and maturity at the time of harvest. This heterogeneity complicates the comparison of extraction efficiencies across studies and hampers the establishment of standardized protocols.

Solvent choice plays a crucial role in the extraction process, impacting both the yield and stability of psilocybin and psilocin. While methanol is widely used due to its strong affinity for polar compounds, it is also highly volatile and requires careful handling to prevent degradation. Additionally, psilocin is particularly susceptible to oxidation, leading to potential losses during extraction and storage. Acidified solvents, such as methanol with acetic acid or hydrochloric acid, have been proposed to enhance stability, but their effect on extraction efficiency varies between studies, further complicating reproducibility.

Operational parameters such as temperature, extraction time, solvent-to-material ratio, and agitation methods significantly impact psilocybin yields. However, there is a lack of consensus on optimal conditions, with different studies employing varied methodologies. For instance, ultrasonic-assisted extraction (UAE) has been reported as the most efficient method for maximizing yields, but discrepancies remain regarding optimal ultrasound frequency, extraction duration, and solvent combinations. Similarly, vortex agitation and maceration produce variable results, often requiring extended processing times to achieve comparable yields.

The quantification of extracted psilocybin and psilocin is another critical factor affecting reproducibility. High-performance liquid chromatography (HPLC) is the most commonly employed analytical technique, but differences in detection methods (e.g., UV vs. mass spectrometry) and calibration standards can introduce inconsistencies. Furthermore, the use of alternative techniques, including thin-layer chromatography (TLC) and gas chromatography–mass spectrometry (GC-MS), complicates direct comparisons between studies. Without standardized analytical protocols, the accuracy and comparability of the reported yields remain questionable.

While laboratory-scale extractions provide valuable insights into method efficiency, their applicability in large-scale production remains limited. The scalability of extraction methods is constrained by factors involving solvent recovery, cost-effectiveness, and regulatory compliance. Techniques that require hazardous solvents or extreme processing conditions may not be feasible for industrial applications, necessitating the development of greener, more sustainable extraction approaches.

To improve reproducibility, future studies should incorporate chemometric approaches, which integrate statistical modeling, multivariate analysis, and machine-learning algorithms to optimize extraction parameters systematically. By analyzing large datasets of extraction conditions and yields, researchers can identify patterns that enhance process efficiency and minimize variability. This approach would facilitate the development of robust, reproducible protocols that are applicable across different research and industrial settings.

The limitations of current extraction techniques underscore the need for standardized methodologies that account for variability in fungal composition, solvent interactions, operational parameters, and analytical procedures. Addressing these challenges through systematic optimization and chemometric approaches will enhance the reproducibility of psilocybin extraction, supporting its broader application in pharmaceutical and clinical research. Future efforts should focus on refining protocols that maximize yield while ensuring consistency, stability, and scalability.

## 4. Conclusions

The present review systematically evaluates the extraction and quantification methods for psilocybin and psilocin from *Psilocybe* mushrooms, highlighting the critical factors influencing yield and efficiency. The most significant finding is that ultrasonic-assisted extraction (UAE) emerges as the most effective technique, particularly when combined with polar solvents like methanol acidified with acetic or hydrochloric acid. This method maximizes yield through cavitation effects, which efficiently disrupt cell walls and enhance compound recovery. Additionally, the choice of mushroom species, particularly *Psilocybe cubensis* and *Psilocybe cyanescens*, significantly impacts extraction yields, with mushroom caps generally containing higher concentrations of psilocybin and psilocin compared to stems.

The review also underscores the importance of optimizing extraction parameters, such as solvent-to-material ratio, temperature, and extraction time. For instance, maintaining a temperature between 20–25 °C and using multiple consecutive extractions can significantly improve yield while minimizing the degradation of thermosensitive compounds like psilocin. High-performance liquid chromatography (HPLC) coupled with UV or mass spectrometry detection is identified as the most reliable analytical method for quantifying these compounds, ensuring precision and reproducibility.

The future implications of this research are substantial. The findings provide a foundation for developing standardized protocols that can enhance the reproducibility and scalability of psilocybin extraction, which is crucial for its potential therapeutic applications. Future studies should explore the use of novel solvents, such as deep eutectic solvents (DESs) and ionic liquids, which may offer improved selectivity and environmental sustainability. Additionally, integrating chemometric approaches, including multivariate analysis and machine learning, could further optimize the extraction conditions by identifying the most influential parameters affecting yield and stability.

In conclusion, this review not only consolidates the current knowledge on psilocybin extraction but also sets the stage for future research aimed at refining methodologies for pharmaceutical-grade production. By addressing the variability in extraction yields and standardizing protocols, this work contributes to the growing body of research supporting the therapeutic potential of psilocybin in treating mental health disorders.

## Figures and Tables

**Table 1:** Main methods and yield obtained for extraction of psilocybin and psilocin from different mushroom species.

			Whole Mushroom		Cap		Stem	
Reference	Species	Methodology	%PSCB	%PSC	%PSCB	%PSC	%PSCB	%PSC
[48,56]	-	Shaking and maceration	0.100–0.760	0.040–0.840	NA	NA	NA	NA
	-	Unrest	0.060–0.160	NI	NI	NI	NI	NI
[49]	*Inocybe aeruginascens*	Vortex agitation	0.012–0.013	0.0005	NI	NI	NI	NI
[49]	*Inocybe calamistrata*	Vortex agitation	ND	ND	NI	NI	NI	NI
[49]	*Inocybe corydalina*	Vortex agitation	0.008–0.028	0–0.0006	NI	NI	NI	NI
[49]	*Agaricus bisporus*	Vortex agitation	ND	ND	NI	NI	NI	NI
[40]	Blue Meanie	Vortex agitation	1140–1220	0.021–0.051	NI	NI	NI	NI
[40]	B-Plus	Vortex agitation	0.860–1.360	0.013–0.031	NI	NI	NI	NI
[47]	*Conocybe cyanopus*	-	0.330–0.55	0.004–0.007	NA	NA	NA	NA
[47]	*Conocybe tenera*	-	ND	ND	NA	NA	NA	NA
[62]	*Copelandia*	Ultrasonic bath	0.080–0.220	0.430–0.760	0.020–0.220	0.360–0.740	0.010–0.390	0.310–0.780
[47]	*Coprinus plicatilis*	-	ND	ND	NI	NI	NI	NI
[40]	Creeper	Vortex agitation	1000–1600	0.017–0.031	NI	NI	NI	NI
[49]	*Gymnopilus dilepis*	Vortex agitation	0.003–0.013	0.0024–0.0063	NI	NI	NI	NI
[47]	*Gymnopilus spectabilis*	-	ND	ND	NI	NI	NI	NA
[44,45]	Orange *Hypholoma*	Maceration	0.930	0.021	NA	NA	NA	NA
			0.970–0.990	ND	NA	NA	NA	NA
[61]	Bloodless	Shaker	ND	ND	NA	NA	NA	NA
[47]	*Marasmius oreades*	-	ND	ND	NA	NA	NA	NA
[61]	*Panaeolus acuminatus*	Shaker	ND	ND	NA	NA	NA	NA
[50]	Black panther	Maceration	ND	ND	NA	NA	NA	NA
[49]	Belted *panaeolus*	Vortex agitation	0.011–0.158	0.0007–0.0257	NA	NA	NA	NA
[55,60]	*Panaeolus cyanescens*	Ultrasonic bath	0.020–1.150	0.140–0.90	NI	NI	NI	NI
			ND	NI	NI	NI	NI	NI
[45,47,49]	*Panaeolus foenisecii*	Vortex agitation	ND	ND	NI	NI	NI	NI
		NI	ND	ND	NI	NI	NI	NI
		Maceration	0.680–0.730	ND	NI	NI	NI	NI
[49]	*Panaeolus olivaceus*	Vortex agitation	ND	ND	NI	NI	NI	NI
[49,50]	*Panaeolus papilionaceus*	Vortex agitation	ND	ND	NI	NI	NI	NI
		Maceration	ND	ND	NI	NI	NI	NI
[47,49]	*Panaeolus rickenii*	-	ND	ND	NA	NA	NA	NA
		Maceration	ND	ND	NA	NA	NA	NA
[50]	*Panaeolus sphinctrinus*	Maceration	ND	ND	NA	NA	NA	NA
[50,65]	*Panaeolus subbalteatus*	Maceration	1.100–1.300	NA	2.900–4.130	NA	1.100–1.900	NI
		Ultrasonic bath	0.153–0.176	0.012–0.014	NI	NI	NI	NI
[49,51,66]	*Pholiotina cyanopus*	Vortex agitation	0.000–0.086	0.000–0.062	NI	NI	NI	NI
		Ultrasonic bath	0.820–0.980	0.160–0.180	NI	NI	NI	NI
		Ultrasonic bath	0.820–0.980	0.160–0.180	NI	NI	NI	NI
[49]	American *Pluteus*	Vortex agitation	0.117–0.243	0.012–0.035	NI	NI	NI	NI
[49]	*Pluteus glaucotinctus*	Vortex agitation	0.194	0.0013	NI	NI	NI	NI
[47,49,61]	*Pluteus salicinus*	-	0.031–0.135	0.037–0.070	NI	NI	NI	NI
		Vortex agitation	0.350	0.011	NO	NO	NO	NO
		Shaker	ND	ND	NO	NO	NO	NO
[46]	*Psathyrella foenisecii*	Agitation	0.150–0.850	0.00–0.590	NO	NO	NO	NO
[47]	*Psathyrella multipedata*	-	ND	ND	YES	YES	YES	YES
[47]	*Psathyrella velutina*	-	ND	ND	NI	NI	NI	NI
[49]	*Psilocybe*	Vortex agitation	0.051–0.189	0.149–0.205	NI	NI	NI	NI
[47]	*Psilocybe atrobrunnea*	-	ND	ND	NI	NI	NI	NI
[61,64]	*Psilocybe bohemica*	Shaker	0.306–0.622	0.318–0.884	NA	NA	NA	NA
			0.250–1.150	0.000–0.020	NA	NA	NA	NA
[49]	*Psilocybe aerulescens*	Vortex agitation	0.022–0.031	0.034–0.041	NA	NA	NA	NA
[49]	*Psilocybe aureulipes*	Vortex agitation	0.223–0.567	0.050–0.028	NA	NA	NA	NA
[35,49,53,55,59,60,62]	*Psilocybe cubensis*	Vortex agitation	0.065–0.351	0.021–0.534	NI	NI	NI	NI
		Ultrasonic bath	0.000–1.070	0.010–0.230	NI	NI	NI	NI
			0.370–1.300	0.140–0.420	0.440–1.350	0.170–0.780	0.050–1.270	0.090–0.900
			0.00048	NI	NI	NI	NI	NI
		Agitation	NI	NI	0.42	0.168	NI	NI
		Ultrasonic bath and mixer	0.923–1.379	0.060–0.192	NI	NI	NI	NI
		-	0.010–1.000	NI	NI	NI	NI	NI
[49,53,61]	*Psilocybe cyanescens*	Vortex agitation	0.234–1.380	0.041–1.002	NI	NI	NI	NI
		Shaker	0.455–1.565	0.450–1.588	NI	NI	NI	NI
		Ultrasonic bath and mixer	1606–3422	0.621–1.767	NI	NI	NI	NI
[49]	*Psilocybe fimetaria*	Vortex agitation	ND	ND	NI	NI	NI	NI
[49]	*Psilocybe fuscofulva*	Vortex agitation	ND	ND	NI	NI	NI	NI
[49]	*Psilocybe medullosa*	Vortex agitation	0.014–0.100	0.000–0.005	NI	NI	NI	NI
[49]	*Psilocybe mexicana*	Vortex agitation	0.328–0.393	0.194–0.197	NI	NI	NI	NI
[49,57]	*Psilocybe ovoideocystidiata*	Ultrasonic bath	NI	NI	1020–1790	0.140–0.460	0.170–0.190	0.040
		Vortex agitation	0.091–0.717	0.003–0.546	NI	NI	NI	NI
[58]	*Psilocybe pelliculosa*	Ultrasonic bath	NI	NI	1020–1790	0.140–0.460	0.170–0.190	0.040
[49,55,58,61,64,66]	*Psilocybe semilanceata*	Vortex agitation	0.128–1.142	0.003–0.062	NI	NI	NI	NI
		Shaker	0.300–0.322	0.146–0.158	NI	NI	NI	NI
		Ultrasonic bath	0.010–0.910	0.010–0.900	NI	NI	NI	NI
		Ultrasonic bath	1340–1580	0.228–0.252	NI	NI	NI	NI
		Shaker	0.250–1.150	0.000–0.020	NI	NI	NI	NI
		Agitation	0.120–0.360	NI	NI	NI	NI	NI
[49,61]	*Psilocybe serbica*	Vortex agitation	0.156–0.396	0.021–0.381	NI	NI	NI	NI
		Shaker	0.094–0.820	0.310–0.370	NI	NI	NI	NI
[49]	*Psilocybe serbica* var. *arcana*	Vortex agitation	0.0002–0.878	0.041–0.792	NI	NI	NI	NI
[49]	*Psilocybe serbica* var. *bohemica*	Vortex agitation	0.155–1.554	0.003–0.248	NA	NA	NA	NA
[49]	*Psilocybe serbica* var. *moravica*	Vortex shaking	0.565–1.416	0.006–0.038	NA	NA	NA	NA
[44,45,49]	*Psilocybe subaeruginosa*	Vortex shaking	0.010–0.019	0.008–0.033	NA	NA	NA	NA
		Maceration	1410	0.038	NA	NA	NA	NA
			1.070–1.120	0.011–0.019	NA	NA	NA	NA
[47]	*Psilocybe subcoprophila*	-	ND	ND	NA	NA	NA	NA
[52]	*Psilocybe subcubensis*	Ultrasonic bath	NA	NA	0.86	0.02	0.8	0.03
[53,55]	*Psilocybe tampanensis*	Ultrasonic bath	0.000–0.190	0.010–0.030	NA	NI	NI	NI
		Ultrasonic bath and mixer	0.057–0.181	0.015–0.101	NI	NI	NI	NI
[49,54]	*Psilocybe zapotecorum*	Vortex agitation	0.902–0.965	0.029–0.037	NI	NI	NI	NI
		Ultrasonic bath	0.110–0.260	0.038–0.650	0.190–0.310	0.110–0.510	0.080–0.240	0.030–0.220
[63]	*Psilocybe*	Vortex agitation	0.950–1.030	NI	NI	NI	NI	NI
[49]	*Stropharia aeruginosa*	Vortex agitation	ND	ND	NI	NI	NI	NI
[47]	*Stropharia semiglobara*	-	ND	ND	NI	NI	NI	NI
[60]	Alleged *Psilocybe cubensis*	Ultrasonic bath	0.0008	NI	NI	NI	NI	NI
[40]	Texas Yellow	Vortex agitation	1000–1160	0.019–0.028	NI	NI	NI	NI
[40]	Thai *Cubensis*	Vortex agitation	0.740–0.880	0.050–0.090	NI	NI	NI	NI

NA: not analyzed, ND: not detected; NI: not informed.

**Table 2:** Parameters and extraction methods of psilocybin and psilocin in mushrooms.

Reference	Methodology	Mushroom Form	Spraying Technique	Solvent Type	Proportion (*m*/*v*)	Time (h)	Number of Extractions	Temperature (°C)	Performance	
									PSCB (%)	PSC(%)
[56]	Agitation	Dust	Mill	Methanol	(1:50)	one night	1	NI	0.10–0.76	0.04–0.84
[48]	Agitation and maceration	NI	NI	Methanol	(1:10)	0.75 and one night	1	NI	0.06–0.16	NI
[49]	Vortex agitation	Dust	Mortar	Methanol/acetic acid + Methanol	(1:100)	1	NI	20	0.0002–1.5543	0–1.0018
[40]	Vortex Agitation	Dust	Mill	Methanol/acetic acid	(1:100)	0.5	2	NI	0.74–1.6	0.013–0.051
[47]	-	NI	NI	Methanol/ammonium nitrate	(9:1)	NI	2	NI	ND–0.55	ND–0.011
[62]	Ultrasonic bath	Dust	Mortar	Methanol	(1:100)	0.5	1	NI	0.08–0.22	0.43–0.76
[44]	Maceration	NI	NI	Methanol/sodium polyphosphate	(1:100)	one night	1	NI	0.93–1.41	0.021–0.038
[45]	Maceration	Dust	Mill	Methanol	(1:1)	24	1	NI	0.68–1.12	0.011–0.019
[61]	Shaker	Dust	Mortar	Methanol	(1:50)	0.5	1	NI	0.094–1.565	0.146–1.588
[50]	Maceration	Dust	Mortar	Water/ethanol	(1:100)	One night	2	20–25	1.1–1.9	ND
[55]	Ultrasonic bath	Dust	NI	Methanol	(1:90)	2	1	50	0–1.15	0.01–0.9
[60]	Ultrasonic bath	Dust	Mill	Methanol/disodium 3-indoxyl phosphate	(1:1000)	0.5	2	<50	0.001–1.30	0.014–0.42
[65]	Ultrasonic bath	Dust	-	Methanol/water	(1:20)	0.25	1	NI	0.153–0.176	0.012–0.014
[51]	Ultrasonic bath	Dust	Mortar	Methanol	(1:100)	3	1	NI	0.82–0.98	0.16–0.18
[66]	Ultrasonic bath	NI	NI	Methanol	(1:100)	3	1	NI	0.82–1.58	0.16–0.252
[46]	Agitation	Dust	Mill	Methanol	(1:28)	12	1	20–25	0.15–0.85	0–0.59
[64]	Agitation	NI	NI	Methanol	(1:100 and 1:10)	16	1	25	0.25–1.15	0–0.02
[53]	Ultrasonic bath and mixer	Powder and whole	Mill	Methanol/hydrochloric acid	(1:1000) and NI	1	1	20–25	0.00048–3.422	0.015–1.767
[35]	Ultrasonic bath	Dust	Mortar	Chloroform	(1:15)	1	1	NI	NI	NI
[59]	Agitation	Dust	Mortar	Methanol	(1:100)	24	1	20–25	0.923–1.379	0.06–0.192
[57]	Ultrasonic bath + vortex	Dust	Mill	Methanol/water	(1:10)	0.25 + 0.5	1	NI	NI	NI
[58]	Agitation	Dust	Mill	Methanol	NI	20	1	20–25	0.08–0.36	NI
[52]	Ultrasonic bath	Dust	Mortar	Chloroform	(1:20)	1	1	NI	NI	NI
[54]	Ultrasonic bath	Dust	Mortar	Methanol and Methanol/water + formic acid	(1:33)	0.17	3	20–25	0.11–0.26	0.038–0.65
[63]	Vortex agitation + ultrasonic bath	Dust	Mill	Methanol	(1:200)	NI + 0.083	2	NI	0.95–1.03	NI

ND: not detected; NI: not informed.

## Appendix A. Construction of the Systematic Review

**Table A1:** Strings and databases used to retrieve the studies.

Database	String	Results	Search Date
Scopus	(ALL ((psilocybin OR psilocibina OR psilocin OR psilocina)) AND ALL ((extraction OR extração OR extracción OR identification OR identificação OR identificación OR characterization OR caracterização OR descripción OR quantification OR quantificação OR cuantificación)))	3882	11 September 2024
Science Direct	(Psilocybin OR Psilocin) AND (Extraction OR Identification OR Identificação OR Characterization OR Quantification)	3065	11 September 2024
Sage Journal	(Psilocybin OR Psilocibina OR Psilocin OR Psilocina) AND (Extraction OR Extração OR Extracción OR Identification OR Identificação OR Identificación OR Characterization OR Caracterização OR Descripción OR Quantification OR Quantificação OR Cuantificación)	818	11 September 2024
Web of Science	ALL = ((Psilocybin OR Psilocibina OR Psilocin OR Psilocina) AND (Extraction OR Extração OR Extracción OR Identification OR Identificação OR Identificación OR Characterization OR Caracterização OR Descripción OR Quantification OR Quantificação OR Cuantificación))	195	11 September 2024
BVS	((Psilocybin OR Psilocibina OR Psilocin OR Psilocina)) AND ((Extraction OR Extração OR Extracción OR Identification OR Identificação OR Identificación OR Characterization OR Caracterização OR Descripción OR Quantification OR Quantificação OR Cuantificación))	253	11 September 2024
LILACS	((Psilocybin OR Psilocibina OR Psilocin OR Psilocina)) AND ((Extraction OR Extração OR Extracción OR Identification OR Identificação OR Identificación OR Characterization OR Caracterização OR Descripción OR Quantification OR Quantificação OR Cuantificación))	253	11 September 2024
PubMed	(Psilocybin OR Psilocibina OR Psilocin OR Psilocina) AND (Extraction OR Extração OR Extracción OR Identification OR Identificação OR Identificación OR Characterization OR Caracterização OR Descripción OR Quantification OR Quantificação OR Cuantificación)	332	11 September 2024
Embase	(‘psilocybin’/exp OR psilocybin OR psilocibina OR ‘psilocin’/exp OR psilocin OR psilocina) AND (‘extraction’/exp OR extraction OR extração OR extracción OR ‘identification’/exp OR identification OR identificação OR identificación OR ‘characterization’/exp OR characterization OR caracterização OR descripción OR ‘quantification’/exp OR quantification OR quantificação OR cuantificación)	293	11 September 2024
Cochrane	(Psilocybin OR Psilocibina OR Psilocin OR Psilocina) AND (Extraction OR Extração OR Extracción OR Identification OR Identificação OR Identificación OR Characterization OR Caracterização OR Descripción OR Quantification OR Quantificação OR Cuantificación)	39	11 September 2024
Engineering Village	(Psilocybin OR Psilocin) AND (Extraction OR Identification OR Identificação OR Characterization OR Quantification)	22	11 September 2024

**Table A2:** Inclusion and exclusion criteria for studies.

Inclusion Criteria (I)	Exclusion Criteria (E)
We accept articles with only psilocybin or psilocin.	We reject articles with a score less than or equal to 5.
We accept articles that contain the words psilocybin or psilocin in the title, abstract, and/or keywords.	We reject articles that are not experimental.
	We reject articles that do not deal with the extraction, identification, quantification, or chromatography of psilocybin and psilocin.
	We reject articles containing biological/in vivo/clinical tissues/fluids analysis.

## Appendix B. Quality Analysis of Selected Studies

**Table A3:** Criteria for quality analysis.

1	Was the aim of the study clearly defined and relevant to psilocybin extraction?
2	Were the extraction methodologies (e.g., solvents, time, temperature) described in sufficient detail?
3	Was the sample preparation process adequately explained (e.g., drying, grinding, storage conditions)?
4	Were the solvents and reagents properly justified and aligned with the goals of the extraction process?
5	Was the extraction efficiency (yield of psilocybin/psilocin) clearly reported?
6	Were the analytical methods (e.g., HPLC, TLC, GC-MS) for psilocybin quantification validated and appropriate?
7	Was the reproducibility and consistency in the replication of the experiments?
8	Were the data and results presented in a clear, organized, and statistically valid manner?
9	Were the limitations or challenges of the extraction or quantification process discussed?
10	Were the conclusions supported by the data and consistent with the study’s objectives?
11	Does the study discuss potential sources of bias and how they were addressed during the experimental process?
12	Was the study’s contribution to the field clearly articulated and placed within the context of existing literature?
